# Immune Adaptation of Colorectal Cancer Stem Cells and Their Interaction With the Tumor Microenvironment

**DOI:** 10.3389/fonc.2020.588542

**Published:** 2020-11-18

**Authors:** Chun-Chi Lin, Tsai-Tsen Liao, Muh-Hwa Yang

**Affiliations:** ^1^ Institute of Clinical Medicine, National Yang-Ming University, Taipei, Taiwan; ^2^ Faculty of Medicine, School of Medicine, National Yang-Ming University, Taipei, Taiwan; ^3^ Division of Colorectal Surgery, Department of Surgery, Taipei Veterans General Hospital, Taipei, Taiwan; ^4^ Graduate Institute of Medical Sciences, College of Medicine, Taipei Medical University, Taipei, Taiwan; ^5^ Cell Physiology and Molecular Image Research Center, Wan Fang Hospital, Taipei Medical University, Taipei, Taiwan; ^6^ Cancer Progression Research Center, National Yang-Ming University, Taipei, Taiwan; ^7^ Division of Medical Oncology, Department of Oncology, Taipei Veterans General Hospital, Taipei, Taiwan

**Keywords:** colorectal cancer stem cells, immune evasion, metastasis, tumor microenviroment, immunotherapy

## Abstract

Metastasis is the primary cause of death in colorectal cancer (CRC) patients. Emerging evidence has shown that CRC stem cells (CRCSCs) play a significant role in metastatic dissemination and tumor recurrence. However, strategies for targeting CRCSCs are limited because CRCSCs are resistant to therapeutic interventions and because the tumor microenvironment (TME) provides a supportive niche. Moreover, growing evidence highlights the critical role of CRCSCs in immune adaptation and modulation of the TME. CRCSCs escape immune surveillance by avoiding recognition by the innate immune system and shaping the TME through exosomes, cytokines, and chemokines to generate an immunosuppressive niche that facilitates cancer progression. In this review, we summarize studies investigating the immunomodulatory properties of CRCSCs and their underlying mechanisms in order to improve the efficacy of treatment strategies against advanced CRC.

## Introduction

Colorectal cancer (CRC) is one of the most common and deadly cancers worldwide. The global CRC burden is expected to have increased by 60% by 2030 (1, 2). Improvements in the diagnosis, screening, and treatment of CRC have significantly increased the long-term survival rates of patients with early-stage disease. However, outcomes of patients with advanced-stage disease are still unsatisfactory (3). Although surgical resection is the main strategy for treating primary CRC, surgery alone is insufficient in patients with advanced-stage disease, and combination treatments including chemotherapy, targeted therapy, radiation therapy, and immunotherapy are mandatory for combating disseminated CRC. However, the overall survival (OS) of metastatic (m)CRC patients is dismal, with a 5-year OS rate of only 13.5% (4). Approximately 20% of CRC patients have synchronous metastases at initial diagnosis, which most commonly occur in the liver, and up to 60% of patients develop distant metastases within 5 years (5–7).

Cancer stem cells (CSCs) are a minor subpopulation of tumor cells that have self-renewal, tumor-initiation, therapeutic-resistance, and clonal long-term repopulation abilities (8–10). CSCs are considered the driving force behind cancer progression and metastasis. Therefore, targeting CSCs provides a therapeutic opportunity for managing metastatic disease. The existence of CSCs in CRC is supported by lineage-tracing experiments (11–13). In CRC, CSCs are generally defined by increased expressions of intestinal SC (ISC) markers, including leucine-rich repeat-containing G-protein coupled receptor 5 (LGR5), cluster of differentiation 24 (CD24), CD29, CD44, and CD133 (14–18). Functional assays examining their self-renewal ability, including serial replication of tumorspheres *in vitro* and serial passaging of bulk tumor cells *in vivo*, are also important for characterizing colorectal CSCs (CRCSCs).

Previous studies indicated that CSCs reside in cellular niches with a favorable tumor microenvironment (TME). These niches protect CSCs from immune surveillance and apoptosis and help them maintain their plasticity (19, 20). CSC plasticity is crucial for enduring environmental stresses and increasing the chance of successfully metastasizing. Moreover, CSCs can modulate their immunological profile, such as increasing expressions of human leukocyte antigen (HLA) class I molecules and programmed death ligand-1 (PD-L1), to escape immune surveillance, which enriches the CSC subpopulation in tumors (21, 22). The crosstalk between CSCs and the TME influences the response to treatment and metastasis. Therefore, an extensive understanding of the interplay between CRCSCs and the TME is warranted so that strategies can be developed to eradicate CRCSCs.

## CRCSCs in Carcinogenesis and Metastasis

The accumulation of both genetic and epigenetic changes triggers colorectal carcinogenesis by transforming colon epithelial cells into heterogeneous adenoma-carcinoma cells (23). Mutations in driver oncogenes and tumor-suppressor genes, such as adenomatous polyposis coli, tumor protein 53, Kirsten rat sarcoma (*KRAS*), and phosphatidylinositol-4,5-bisphosphate 3-kinase catalytic subunit alpha, are present in 81, 60, 43, and 18% of sporadic CRC cases, respectively (24). These mutations promote the transformation of normal intestinal epithelial cells into adenomas, invasive carcinomas, and eventually metastatic tumors (25, 26). Experimental mouse models provide a useful tool to investigate how mutations in these genes affect the regulation of carcinogenesis and metastasis in CRC. For example, crossing APC-deficient mice with mice harboring other driver mutations enhances adenocarcinoma transformation without metastasis (27). Interestingly, transplantation of tumor-derived organoids from these crosses enhances the likelihood of metastasis (27–29). Those results imply that *ex vivo* organoid culture provides selective pressure that subsequently enriches the CSC population to confer a survival advantage and promote metastasis.

Interestingly, increasing evidence indicates that in mCRC, the initial location of the primary tumor is correlated with outcomes. For primary CRCs, tumors in the cecum to the transverse colon are defined as right-side colon cancers (RCCs), whereas those in the splenic flexure to the rectum are known as left-side colon cancers (LCCs). RCCs have a significantly worse prognosis than do LCCs (30–32). In a phase III CALGB/SWOG 80405 trial, different primary origin sites reflected different treatment responses in patients with mCRC. For example, patients with mCRC from RCCs had prolonged progression-free survival when treated with bevacizumab compared to those treated with cetuximab as first-line treatment. Conversely, patients with mCRC from LCCs had longer OS and better overall response rates than those treated with bevacizumab (33, 34). Zhang et al. (35) indicated that RCCs and LCCs had different tumor immunological profiles. RCCs are characterized by increased infiltration of immune cells with enhanced cytotoxic functions, interferon (INF)-γ signatures, and vascular endothelial growth factor (VEGF)-α (VEGFA) and decreased levels of activated cluster of differentiation 8^+^ (CD8^+^) T-cells, T helper cell type 1 (Th1) cells, and protein release factor 1 (PRF1) expressions. Therefore, patients with RCCs respond well to bevacizumab, a humanized anti-VEGF monoclonal antibody that can neutralize VEGFA. However, LCCs are associated with CD56^bright^ natural killer (NK) cells. Cetuximab can bind the Fc receptor, FcγRIII (CD16), on NK cells, inducing antibody-dependent cell-mediated cytotoxicity. This releases cytotoxic granzyme-containing granules, and INF-γ secretion subsequently kills tumor cells (36). Intriguingly, expressions of stemness markers, such as ATP-binding cassette sub-family G member 2 and the POU family of transcription factors, class 5, factor 1, are associated with RCCs. As with CSCs, RCC cells are difficult to eradicate, and relapse and metastasis are common in RCCs (37). These studies indicate that stemness properties and tumor immunological profiles may lead to different treatment responses and outcomes of CRC.

Different studies showed that CSCs in distinct cancer types harbor unique markers. CRCSCs exhibit characteristics that are similar to ISCs (38). CRCSCs and ISCs express similar markers, such as LGR5 (14, 17) and CD44 (16), and share several important signaling pathways, including the WNT, transforming growth factor (TGF)-β, hedgehog, and Notch pathways (39–41). The depletion of Lgr5^+^ cells in CRC restricts primary tumor growth, and such tumors are incapable of forming distant metastases. Therefore, Lgr5^+^ CSCs are essential for metastasis. de Sousa e Melo et al.’s group (29) also showed that Lgr5^+^ CSCs are critical for the formation and maintenance of liver metastases. However, Fumagalli et al. (42) recently showed that the majority of CRC metastases are seeded by Lgr5^–^ cells and reestablish a cellular hierarchy that gives rise to Lgr5^+^ cells. That study reinforced the concept of cancer cell plasticity and also indicated that plasticity is crucial for both primary and metastatic tumor growth. A hybrid epithelial-mesenchymal state may offer a more-plastic status for cancer cells to adapt to the stressful environment they experience during the metastatic process (43).

The consensus molecular subtype (CMS) classification is widely used to classify primary CRC into four subtypes based on transcriptomic profiles. CMS1 (microsatellite instability (MSI)-immune, 14% of patients) includes tumors with high MSI, CMS2 (canonical, 37% of patients) consists of chromosomal unstable tumors, CMS3 (metabolic, 13% of patients) comprises tumors with *KRAS* mutations and metabolic dysregulation, and CMS4 (mesenchymal, 23% of patients) includes tumors with a mesenchymal phenotype and CSC-like subtype. Tumors with mixed features (13% of patients) possibly represent a transition phenotype or intratumoral heterogeneity (44). Becht et al. (45) demonstrated that the CMS subgroups and microenvironmental signatures are highly correlated. CMS1 exhibits increased infiltration of activated CD8^+^ cells, NK cells, and T-cell-attracting chemokines, which were shown to be correlated with a better prognosis (45–47). In contrast, CSC-like CMS4 expresses high levels of the myeloid chemokine, CCL2, complement components, angiogenic factors, and other immunosuppressive factors. This leads to a highly vascularized and inflammatory tumor with a high density of cancer-associated fibroblasts. Therefore, immune infiltrates of CMS1 and CMS4 display divergent functional orientations. CMS1 tumors are associated with favorable outcomes since they express immunologic constants of rejection genes, whereas CMS4 tumors have an unfavorable, inflamed immune phenotype and are associated with worse survival (45). Therefore, patients with CMS1 tumors may theoretically benefit from immune checkpoint inhibitors (ICIs), whereas those with the CMS4 subtype would be suitable for strategies combining inhibitors of immunosuppressive components, such as transforming growth factor (TGF)-β, regulatory T cells (Tregs), myeloid-derived suppressor cells (MDSCs), and immune checkpoint molecules (48).

Notably, treatment decisions for patients with metastatic disease are based on the molecular characteristics of the primary resected tumor. To effectively treat recurrent/metastatic CRC, the major question is whether the primary CMS reflects the gene signature of metastatic sites. Recently, Piskol et al. (49) applied a NanoString-based CMS classifier and indicated that tumor-intrinsic features, such as genetic alterations and tumor-specific gene expressions, are maintained during CRC progression in orthotopic models. Therefore, transcriptomes of tumor cells do not change during metastatic evolution. However, changes in extrinsic factors, such as the environmental composition (e.g., stromal content), may explain the discordance of CMS subtypes in primary and metastatic samples (49).

## CRCSCs and the TME

The TME is composed of mesenchymal cells, tumor-infiltrating immune cells (TIICs), endothelial cells, extracellular matrix (ECM), and inflammatory mediators (50).

CRCSCs can modulate the TME through the secretion of tumor-associated exosomes (TAEs). Exosomes are cell-derived vesicles with a diameter ranging 30~100 nm that serve as important mediators for intercellular communication under both physiological and pathological conditions (51). The diverse molecules carried inside exosomes, such as proteins, enzymes, and nucleic acids, have different functions and are involved in the establishment of the pre-metastatic niche (51, 52). For example, exosomal integrins determine organotropic metastasis. The exosomal integrins, α_6_β_4_ and α_6_β_1_, are associated with lung metastasis, whereas the exosomal integrin, α_v_β_5_, is linked to liver metastasis (53). Rana et al. (54) showed that TAEs target non-transformed cells in premetastatic organs and modulate premetastatic organ cells predominantly through transferred micro (mi)RNAs. We recently showed that CRCSCs secrete exosomal *miR-146* to promote stem-like properties and tumorigenicity by targeting Numb in recipient colon cells. Notably, in clinical samples, *miR-146a* ^High^/Numb^Low^ tumors had an increased number of tumor-infiltrating CD66^+^ neutrophils and a decreased number of tumor-infiltrating CD8^+^ T cells, indicating an immunosuppressive TME (55). CRCSC-secreted exosomes also mediate interleukin (IL)-1β expression in neutrophils, thus prolonging neutrophil survival and inducing a protumoral phenotype. Furthermore, CRCSC-secreted C-X-C motif chemokine (CXCL)-1 and CXCL2 promote migration of neutrophils as a positive feedback mechanism for their stem-like function (56). CD44v6-positive CRCSCs also assist in cancer colonization, invasion, and metastasis. CD44v6 serves as a binding site for Fas, thus preventing Fas-mediated cell death by CD8^+^ T cells (57).

Activated signaling pathways in CRCSCs not only enhance CSC properties but also shape the TME as a proper niche for metastasis. For example, activation of the NOTCH1 signaling pathway plays a role in CRC stemness, creates a TME associated with worse CRC subtypes, and drives metastasis through TGF-β-dependent neutrophil recruitment (58). CSCs also secrete cytokines and chemokines to regulate the immune response and shape a protumoral TME (59–61). For example, CSC-secreted CXCL12 interacts with CXCR4 to inhibit CRC growth, survival, and migration (62). Blocking the CXCL12-CXCR4 interaction reduces CD44v6 expression in CRCSCs (63). CRCSCs also activate IL-6/STAT3 signaling, and IL-6 is associated with advanced CRC. IL-6 is required for the induction of effector Th17 cells and inhibits the differentiation of Tregs during chronic inflammation. Blocking the IL-6/STAT3 axis diminishes CRC tumor growth in vivo (64, 65).

## Immunomodulation of CRCSCs

TIICs in the TME have dual functions in cancer progression: TIIC-related inflammation facilitates tumorigenesis, and TIICs also harbor antitumor properties when appropriately activated. Cancer cell-secreted factors hijack TIIC functions to promote tumor development and metastasis.

The interplay between cancer cells and host immune cells in the TME has been an attractive topic for cancer research owing to the great success of ICIs in treating advanced cancers. These ICIs include monoclonal antibodies (mAbs) targeting cytotoxic T-lymphocyte-associated antigen-4, programmed death (PD)-1, and PD ligand 1 (PD-L1). PD-1 on tumor-infiltrating lymphocytes interacts with its ligand, PD-L1, on other cells. This interaction blocks T-cell receptor-mediated signal activation, preventing further antigen-mediated T-cell activation. PD-L1 is expressed by many types of cells, including tumor cells, immune cells, epithelial cells, and endothelial cells (66). The US Food and Drug Administration (FDA) has approved ICIs, including the PD-1-blocking mAbs, pembrolizumab and nivolumab, and the PD-L1-targeted mAb, atezolizumab (67), for different types of cancer. Unfortunately, ICIs only benefit a small subset of mCRC patients, mainly those with mismatch repair-deficient tumors. These tumors are associated with a high level of microsatellite instability (MSI) and a high tumor mutational burden. Interestingly, growing evidence also showed that PD-L1 expression is dramatically increased in CSCs (68, 69), which not only contributes to immune evasion but also promotes stem-like properties (70–72). Mechanistically, the epithelial-to-mesenchymal transition (EMT) enriches PD-L1 in CSCs through the EMT/β-catenin/STAT3/PD-L1 signaling axis (22). In hepatocellular carcinoma, IL-6 was shown to activate phosphorylation of PD-L1 on Tyr112 by Janus kinase 1 (JAK1) and then recruit the endoplasmic reticulum-associated *N*-glycosyltransferase STT3A to maintain PD-L1 stability (73). In CRCSCs, we recently showed that the epigenetic regulation of the ARID3B/KDM4C axis not only enhances expressions of ISC-specific stemness genes but also promotes PD-L1 expression (74).

Some studies showed that CRCSCs also have low expressions of HLA class I and II molecules and high expressions of immunomodulatory molecules such as IL-4, which inhibit antitumor T cell responses. Conversely, another study showed no difference in HLA class I expressions between CRCSCs and non-CSCs (75). One possible explanation is that established cell lines might not accurately reflect the properties of primary CSCs (76). Moreover, CRCSCs were found to express more ligands for natural killer (NK) cell receptors. Therefore, CRCSCs are more susceptible to freshly purified allogeneic NK cells than are non-CSCs. Lower expression levels of MHC class I also benefit NK recognition and function (77).

## Conclusions

**Figure 1:**
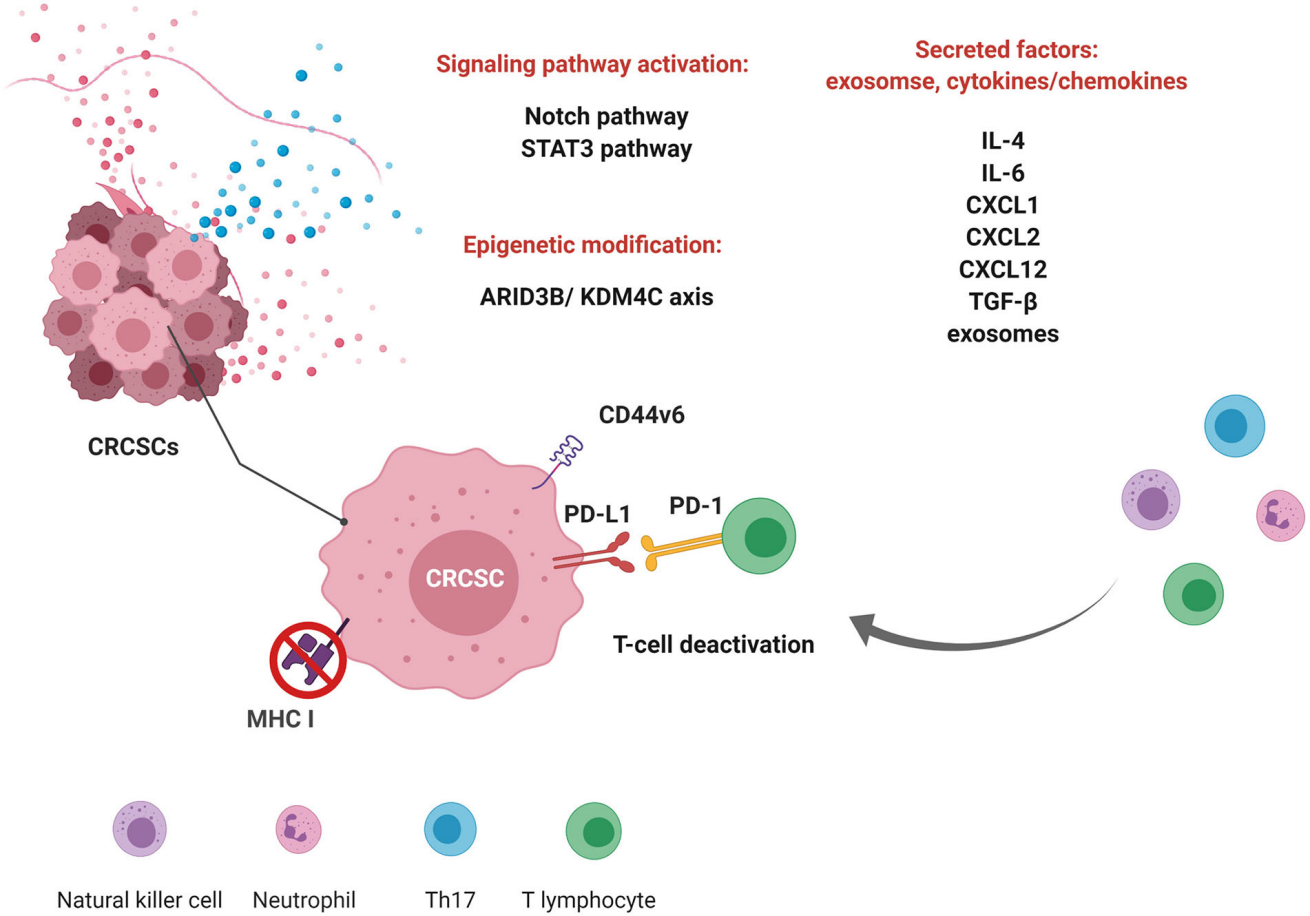
Immunomodulation of colorectal cancer stem cells (CRCSCs) and the interplay between CRCSCs and the tumor microenvironment. Crosstalk between CRCSCs and immune cells through signaling pathways, epigenetic modulation, and secretory factors shapes the tumor microenvironment to promote survival. CRCSCs modulate the expression of antigen presentation molecules (major histocompatibility complex (MHC) I) and surface markers [programmed death ligand 1 (PD-L1) and cluster of differentiation 44v6 (CD44v6)] to inhibit the activities of immune cells. They also secrete factors to modulate the tumor microenvironment. (This figure was created with BioRender.com.).

CSCs are critical for the development of metastasis, which makes them an attractive target for cancer treatment. However, the direct targeting of CSCs has failed because CSCs can regenerate, and non-CSCs can be dedifferentiated into CSCs, both of which are supported by microenvironmental signals that produce stemness-inducing factors. Additionally, cancer cell plasticity can be triggered independently of stemness-inducing factors provided by niches. Because of the complexity of CSCs and the TME (**Figure 1**), further studies on immunomodulatory factors, immunological profiles, and endogenous cellular plasticity are warranted. There are ongoing clinical trials to therapeutically target these CRCSCs combined with TME modulation to improve patient outcomes. For example, the phase I/II study, NCT02176746, is using a CSC-loaded dendritic cell as a vaccine as active immunotherapy for CRCSCs. CSCs are more immunogenic and effective in inducing antitumor immunity. CSC-vaccinated hosts contained high levels of immunoglobulin G (IgG) which was bound to CSCs, resulting in CSC lysis by complement activation. In addition, cytotoxic T-lymphocytes (CTLs) generated from peripheral blood mononuclear cells or splenocytes harvested from CSC-vaccinated hosts were capable of killing CSCs in vitro. CSC-primed antibodies and T cells were capable of selective targeting of CSCs thereby conferring antitumor immunity (78). There is another phase 3 trial, NCT02753127, in which adult patients with previously treated metastatic colorectal cancer are being treated with Napabucasin (BBI-608) combined with 5-fluorouracil, leucovorin, and irinotecan (FOLFIRI). BBI-608 is a small-molecule STAT3 inhibitor that can directly inhibit STAT3-driven signaling activation, a critical regulator of cancer stemness. The standard CRC regimen, 5-**f**luorouracil, **l**eucovorin, and **o**xaliplatin **(**FOLFOX), was evaluated and shown that Tregs were significantly reduced in those with high baseline levels, with no change in relative proportions of CD4, CD8, or NK cells (79, 80). This concise review summarizes the updated connection of CRC from primary and metastatic sites and highlights the importance of the TME in cancer progression. This article reviews the interplay between CRCSCs and the TME. These studies can facilitate improving current treatment modalities and designing innovative strategies for immunotherapeutic approaches to target CSCs.

## Author Contributions

Manuscript writing and final approval were completed by C-CL, T-TL, and M-HY. All authors contributed to the article and approved the submitted version.

## Funding

This work was supported by the Ministry of Science and Technology (MOST 108-2314-B-010-020-MY3 and MOST 108-2320-B-010-008 to M-HY., MOST 109-2636-B-038-001 to T-TL, and MOST 109-2314-B-075-026 to C-CL), the National Health Research Institutes (NHRI-EX109-10919BI and 08A1-MGGP08-037 to M-HY), Taipei Medical University (TMU108-AE1-B25 to T-TL), Taipei Veterans General Hospital (V109C-112 and V108E-006-01 to M-HY), the Featured Areas Research Center Program within the framework of the Higher Education Sprout Project by the Ministry of Education (to M-HY), and the Center of Excellence for Cancer Research granted by the Ministry of Health and Welfare (MOHW109-TDU-B-211-134019 to M-HY).

## Conflict of Interest

The authors declare that the research was conducted in the absence of any commercial or financial relationships that could be construed as a potential conflict of interest.
